# Simultaneous Spectrophotometric Method for Determination of Emtricitabine and Tenofovir Disoproxil Fumarate in Three-Component Tablet Formulation Containing Rilpivirine Hydrochloride

**DOI:** 10.1155/2014/541727

**Published:** 2014-11-16

**Authors:** S. Venkatesan, N. Kannappan

**Affiliations:** Department of Pharmacy, Annamalai University, Tamil Nadu 608002, India

## Abstract

Developing a single analytical method for estimation of individual drug from a multidrug composition is a very challenging task. A complexation, derivatization, extraction, evaporation, and sensitive-free direct UV spectrophotometric method is developed and validated for the simultaneous estimation of some antiviral drugs such as emtricitabine (EMT), tenofovir disoproxil fumarate (TDF), and rilpivirine HCl (RPV) in tablet dosage form by Vierordt's method. The solutions of standard and sample were prepared in methanol. The *λ*_max⁡_ for emtricitabine, tenofovir disoproxil fumarate, and rilpivirine hydrochloride were 240.8 nm, 257.6 nm, and 305.6 nm, respectively. Calibration curves are linear in the concentration ranges 4–12 *μ*g/ml for EMT, 6–18 *μ*g/ml for TDF, and 0.5–1.5 *μ*g/ml for RPV, respectively. Results of analysis of simultaneous equation method were analyzed and validated for various parameters according to ICH guidelines.

## 1. Introduction

**Figure 1:**
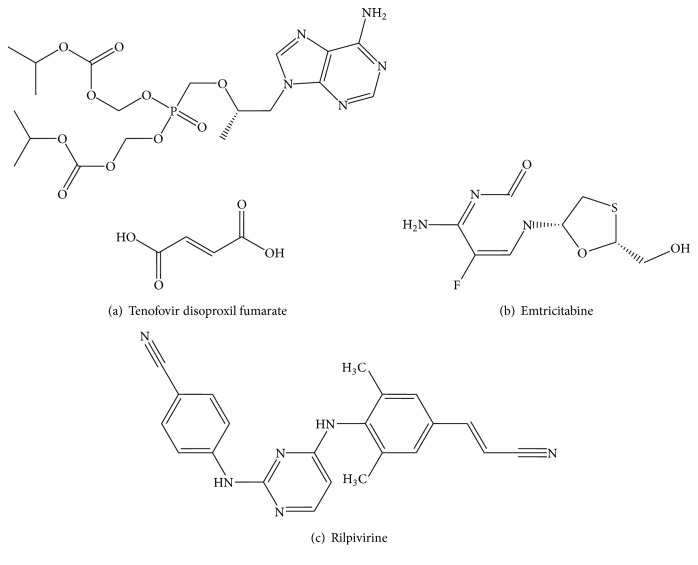
Chemical structure of (a) emtricitabine, (b) tenofovir disoproxil fumarate, and (c) rilpivirine.

Around 33.4 million people were living with HIV in year 2008 and around 2 million people have died in the same year. Highly active antiretroviral therapy (HAART) has brought new hope for those people who live with HIV/AIDS by decreasing the morbidity and mortality among people infected with HIV. Highly active antiretroviral therapy also has improved the quality of life among the people who live with HIV/AIDS. Combination therapy is preferred to be the gold standard for the treatment of AIDS so as to maximize potency, minimize toxicity, and diminish the risk for resistance development and reduction of pill burden to once-daily dosing so as to optimize the patient's compliance and reduce the treatment costs. The nucleoside reverse transcriptase inhibitors and nonnucleoside reverse transcriptase inhibitors as multidrug combinations are effective in the therapy of human immunodeficiency virus (HIV) infection and are used as a part of highly active antiretroviral Therapy, for the treatment of HIV 1, 2 [1]. The daily regimen containing emtricitabine, tenofovir disoproxil fumarate, and rilpivirine HCl is virologically and immunologically effective, well-tolerated, and safe with benefits in the lipid profile in the majority of patients (Figure 1) [2]. It is common practice in HIV treatment to give different drugs to the patient. In order to improve the comfort of the daily intake, manufacturers try to combine several active compounds in one dosage form. In this study a UV spectrophotometric method was developed for tablet containing EMT, TDF, and RPV.

Emtricitabine is a nucleoside reverse transcriptase inhibitor (NRTIs). Chemically it is 5-fluoro-1-(2R, 5S)-[2-(hydroxymethyl)-1,3-oxathiolan-5-yl] cytosine. EMT is the enantiomer of thio analog of cytidine which differs from other cytidine analogs, in that it has fluorine in 5th position.

Tenofovir disoproxil fumarate {9-[(R)-2-[[bis [[isopropoxycarbonyl] oxy] methoxy] phosphonyl] methoxy] propyl] adenine fumarate} is a nucleotide analog reverse transcriptase inhibitor (NRTI) and is used for treating HIV infection in adults, in combination with other antiretroviral agents [3, 4].

Rilpivirine HCl chemical name is benzonitrile 4-[[4-[[4-[(1E)-2-cyanoethenyl]-2,6-dimethylphenyl]amino]-2-pyrimidinyl]amino]hydrochloride. It is a second-generation nonnucleoside reverse transcriptase inhibitor (NNRTI) with higher potency, longer half-life, and reduced side effect profile compared with older NNRTIs, such as efavirenz. It is treated with treatment of HIV-1 infection in conjunction with other antiretroviral [5, 6].

Literature indicates spectrophotometry [7–13], HPLC [14–17], HPTLC [18], and LC/MS/MS [19] methods for determination of TDF individually and in combination with other drugs in pharmaceutical formulations, drug substance, and biological matrices. Similarly for EMT individually and in combination with other drugs by UV [20, 21], HPLC in pharmaceutical formulations, drug substance and biological matrices [22–27], HPTLC, LC/MS/MS [28], and stability indicating liquid chromatographic methods [29] were reported. A detailed literature survey for RPV revealed that few analytical methods are available using spectrophotometric [30], HPLC [31], and HPTLC [32], individually. Literatures are available to show the existence of HPLC method for the triple drug combination of TDF, EMT, and RPV as well [5, 6].

However, no spectrophotometric method has yet been reported for simultaneous estimation of emtricitabine, tenofovir disoproxil fumarate, and rilpivirine HCl in tablet dosage forms. These methods mentioned in the literature, especially the chromatographic techniques, are time-consuming, costly, and require expertise. A simple and accurate UV spectrophotometric method developed can be highly useful for the routine analysis of tablet formulations. Hence, an attempt has been made to develop and validate in accordance with ICH guidelines [33].

## 2. Objective

The main objective of the present study is to a develop simple, precise, accurate, and economical analytical method with a better detector range for simultaneous estimation of three-component tablet formulation by Vierordt's method and to validate the above method as per the ICH guidelines.

## 3. Experimental

### 3.1. Apparatus

A double beam UV-visible spectrophotometer (Shimadzu, 1700), attached to a computer software UV probe 2.0, with a spectral width of 2 nm and pair of 1 cm matched quartz cell, was used.

### 3.2. Materials and Reagents

Authentic samples of emtricitabine (EMT) and tenofovir disoproxil fumarate (TDF) were kindly provided by Aurobindo Pharma Ltd. (Hyderabad, India) while rilpivirine HCl (RPV) was kindly gifted from Strides arco Lab. (Bangalore, India). HPLC grade methanol (S.D fine chemical Ltd., Mumbai, India) was used throughout these experiments. Commercially available tablet dosage forms were assayed in the study Complera/Eviplera Gilead Sciences Inc., Canada, labeled to contain 200 mg EMT, 300 mg TDF, and 25 mg of RPV per tablet.

### 3.3. Study of Spectra and Selection of Wavelength

**Figure 2:**
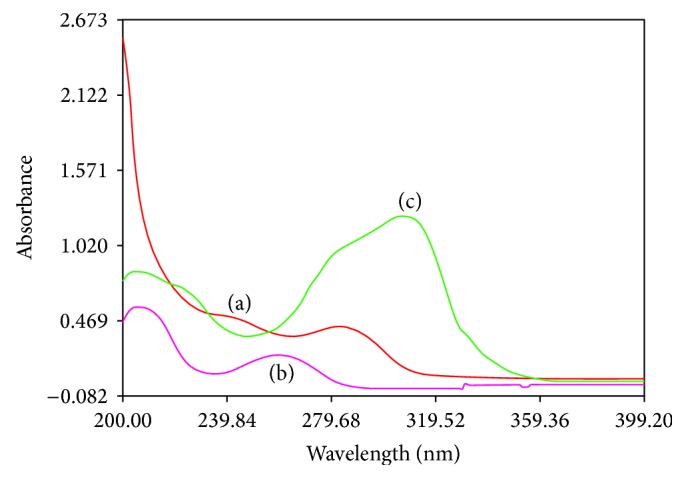
Absorption spectra of 10 *μ*g/mL each of EMT, TDF, and RPV in methanol. (a) UV spectrum of EMT; (b) UV spectrum of TDF; (c) UV spectrum of RPV.

10 *μ*g/mL solution of all three drugs was scanned over the range of 200–400 nm in 1 cm cell against blank and the overlain spectra (Figure 2) were observed. While studying the overlay spectra it was observed that EMT shows maximum absorbance at 240.8 nm, TDF shows maximum absorbance at 257.6 nm, and RPV shows peaks at 305.6 nm, respectively. It was observed that there is no interference for each other at absorbance maxima and spectral characteristics are such that all three drugs can be simultaneously estimated by simultaneous equation method [34].

### 3.4. Standard Solution Preparations

The standard stock solution of EMT, TDF, and RPV was prepared by accurately weighed 20, 30 and 2.5 mg of each drug in 10 mL of volumetric flask separately with methanol. The standard stock solutions were further diluted to get the concentration of 8, 12, and 1 *μ*g/mL of each.

### 3.5. Calibration Curve

**Figure 3:**
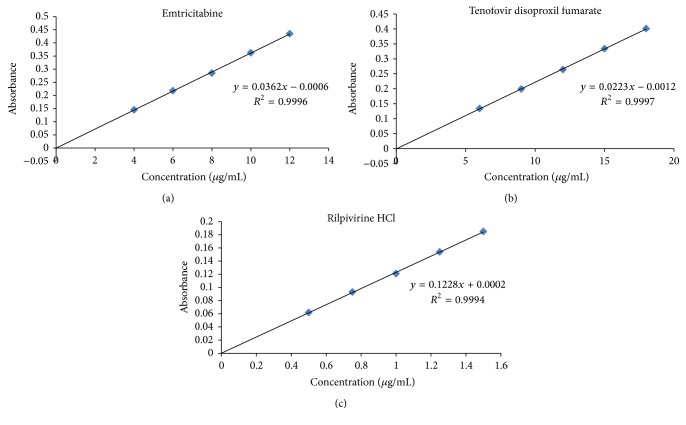
Calibration chart for EMT, TDF, and RPV.

A calibration curve was plotted over a concentration range of 4–12 *μ*g/mL for EMT, 6–18 *μ*g/mL for TDF, and 0.5–1.5 *μ*g/mL for RPV, respectively. For each drug 6 replicates were made by individual weighing (Figure 3).

### 3.6. Simultaneous Equation Method

This method of analysis was based upon the absorption of drugs at wavelength maximum of each other. Three wavelengths of 240.8, 257.6, and 305.6 nm were selected which are the *λ*_max⁡_ of three drugs for the development of the simultaneous equations [35, 36]. The absorbances of EMT, TDF, and RPV were measured and the absorptivity values were determined at all the three selected wavelengths. The concentrations of three drugs in mixture can be calculated using the following equations [37]:

(1) CEMT=A1(ay2az3−az2ay3)−ay1(A2az3−az2A3) +az1(A2ay3−ay2A3)ax1(ay2az3−az2ay3) −ay1(ax2az3−az2ax3)+az1(ax2ay3−ay2ax3)CTDF=ax1(A2az3−az2A3)−A1(ax2az3−az2ax3) +az1(ax2A3−A2ax3)ax1(ay2az3−az2ay3) −ay1(ax2az3−az2ax3)+az1(ax2ay3−ay2ax3),CRPV=ax1(ay2A3−A2ay3)−ay1(ax2A3−A2ax3) +A1(ax2ay3−ay2ax3)ax1(ay2az3−az2ay3) −ay1(ax2az3−az2ax3)+az1(ax2ay3−ay2ax3),

where *C*_EMT_, *C*_TDF_, and *C*_RPV_ are the concentrations of EMT, TDF, and RPV, respectively, in mixture and in sample solutions.

 
*A*_1_, *A*_2_, and *A*_3_  are the absorbances of sample at 240.8, 257.6, and 305.6 nm, respectively,
 
*ax*_1_, *ax*_2_,  and *ax*_3_  are the absorptivity of EMT at 240.8, 257.6 and 305.6 nm, respectively,
 
*ay*_1_, *ay*_2_, and *ay*_3_  are the absorptivity of TDF at 240.8, 257.6, and 305.6 nm respectively,
 
*az*_1_, *az*_2_, and *az*_3_  are the absorptivity of RPV at 240.8, 257.6, and 305.6 nm respectively.

The absorptivity of each solution was calculated by using the following formula [38]:

(2) $$\begin{array}{c} \text{Absorptivity}=\frac{\text{Absorbance}}{\text{concentration}\;\left(\text{gm}/100\;\text{mL}\right)}\;. \end{array}$$

The developed method was validated as per ICH guidelines.

## 4. Results

### 4.1. Specificity

Specificity was studied by measuring the absorbance of EMT, TDF, and RPV individually at 240.8 nm, 257.6 nm, and 305.6 nm against the blank and comparing the absorbance of drugs solutions to the blank. No interference was observed.

### 4.2. Linearity

Linearity of the proposed method was determined by diluting the stock solution to give concentration range of 4–12 *μ*g/mL for EMT, 6–18 *μ*g/mL for TDF, and 0.5–1.5 *μ*g/mL for RPV. The calibration curve was plotted between concentration verses absorbance (Tables 1, 2, and 3).

### 4.3. Accuracy

Accuracy was calculated as the percentage recoveries of blind samples of pure EMT, TDF, and RPV and it indicated the agreement between obtained results and those accepted as true, and detailed results are presented in Table 4. To ascertain the accuracy of the suggested methods, recovery studies were carried out by at three different levels (50%, 100%, and 150% level).

### 4.4. Precision

Intraday (within-day) and Interday (between-day) precision of the proposed methods were determined by estimating the EMT, TDF, and RPV three times on the same day to obtain repeatability and on three different days to obtain the reproducibility. The results are presented in Table 5.

### 4.5. Limits of Detection (LOD) and Quantitation (LOQ)

They were calculated from the standard deviation (d) of the response and the slope of the calibration curve (S) in accordance with the following equations: LOD = 3.3 (d/S) and LOQ = 10 (d/S).

### 4.6. Ruggedness

A study was conducted to determine the effect of variation in analyst to analyst, lab to lab, and instrument to instrument in triplicate measurements as per the assay method. % RSD was calculated for each condition and results are presented in Table 6.

### 4.7. Robustness

As per ICH norms, small, but deliberate, variations by changing the wavelength in ±1 nm from 240.8 nm, 257.6 nm, and 305.6 nm nm and the results are presented in Table 7.

### 4.8. Stability

The stability of EMT, TDF, and RPV standard and sample working solutions in methanol during handling was verified by keeping them at room temperature for 0, 8, and 16 hrs. No significant degradation was observed. The stock solutions were also stable when kept refrigerated at 4°C for at least one week and the absorbance of sample solution in each day was measured. Results are presented in Table 8.

### 4.9. Preparation for Analysis of Tablet Formulation

Twenty tablets were weighed accurately, the average weight of each tablet was determined, and then they were ground to a fine powder. A powder quantity equivalent to 20 mg of EMT, 30 g of TDF, and 2.5 mg of RPV was transferred to a 10 mL volumetric flask and sufficient methanol was added to dissolve it. Then the solutions were sonicated for 15 min. Then final volume was adjusted with methanol and filtered by Whatman filter paper (no. 41). The filtrate was centrifuged at 10,000 RPM for 30 min. Then clear supernatant solutions were transferred to a separate flask without disturbing the sediment. From the clear solution, transfer 0.4 mL of solution to 100 mL volumetric flask. Now the tablet sample solution was scanned in multiphotometric mode and the concentration of all three drugs was obtained from the equation. Results of tablet analysis are reported in Table 9.

## 5. Discussion

The proposed method was validated for precision, accuracy, specificity, linearity and range, limit of detection (LOD) and limit of quantitation (LOQ), robustness, and ruggedness. Validation of the proposed method was carried out in accordance with the International Conference on Harmonization [33] guidelines. The linearity of the calibration plots was confirmed by the high value of the correlation coefficients (*r* ^2^ = 0.9996 for EMT, 0.9997 for TDF, and 0.9994 for RPV). Recovery was in the range of 98–102%; the values of standard deviation and % RSD were found to be <2% showing the high accuracy of the method. The limit of detection and limit of quantification were theoretically calculated which were found to be 0.1392 and 0.4220 for EMT, 0.226 and 0.685 for TDF, and 0.041 and 0.124 for RPV, respectively. Robustness and ruggedness were also carried out and percentage RSD was found to be less than 2.0%. The assay of EMT, TDF, and RPV was found to be 100.42%, 98.63%, and 100.70%. Stability of EMT, TDF, and RPV in methanol was found to be stable up to 7days at room temperature.

## 6. Conclusion

The Vierordt's method has been successfully applied for simultaneous determination of EMT, TDF, and RPV in combined sample solution, and they were found to be accurate, simple, rapid, and precise. Once the equations were constructed, analysis required only measuring the absorbance values of the sample solution at the selected wavelengths followed by few simple calculations. The proposed method was completely validated showing satisfactory data for all the method validation parameters tested. SE method comparably noted to be very efficient in every aspect. Unlike HPLC, by using Simultaneous equation method (UV) the datas can be generated applying simple calculations. So these methods can be easily and conveniently adopted for routine quality control analysis of these cited drugs.

## Figures and Tables

**Table 1:** Absorptivity value for EMT.

Concentration	Absorbance	Absorptivity	Absorbance	Absorptivity	Absorbance	Absorptivity
	*λ*_1_—240.8	*λ*_1_—240.8	*λ*_2_—257.6	*λ*_2_—257.6	*λ*_3_—305.6	*λ*_3_—305.6
4	0.145	362.5	0.113	282.5	0.028	70.0
6	0.218	363.3	0.169	281.6	0.043	71.6
8	0.285	356.2	0.225	281.2	0.057	71.2
10	0.362	362.0	0.281	281.0	0.079	71.0
12	0.435	362.5	0.336	280.0	0.086	71.6

	Absorptivity for *λ*_1_	361.3	Absorptivity for *λ*_2_	281.2	Absorptivity for *λ*_3_	71.1

**Table 2:** Absorptivity value for TDF.

Concentration	Absorbance	Absorptivity	Absorbance	Absorptivity	Absorbance	Absorptivity
	*λ*_1_—240.8	*λ*_1_—240.8	*λ*_2_—257.6	*λ*_2_—257.6	*λ*_3_—305.6	*λ*_3_—305.6
6	0.134	103.3	0.062	233.3	0.000	0.000
9	0.199	363.3	0.169	281.6	0.000	0.000
12	0.264	356.2	0.225	281.2	0.000	0.000
15	0.334	362.0	0.281	281.0	0.000	0.000
18	0.401	362.5	0.336	280.0	0.001	0.555

	Absorptivity for *λ*_1_	361.3	Absorptivity for *λ*_2_	281.2	Absorptivity for *λ*_3_	0.111

**Table 3:** Absorptivity value for RPV.

Concentration	Absorbance	Absorptivity	Absorbance	Absorptivity	Absorbance	Absorptivity
	*λ*_1_—240.8	*λ*_1_—240.8	*λ*_2_—257.6	*λ*_2_—257.6	*λ*_3_—305.6	*λ*_3_—305.6
0.5	0.021	420.0	0.024	480.0	0.062	1240.0
0.75	0.031	413.3	0.038	506.6	0.093	1240.0
1.0	0.041	410.0	0.049	490.0	0.121	1210.0
1.25	0.053	424.0	0.061	488.0	0.154	1232.0
1.50	0.062	413.3	0.072	480.0	0.185	1233.3

	Absorptivity for *λ*_1_	416.1	Absorptivity for *λ*_2_	488.9	Absorptivity for *λ*_3_	1231.0

**Table 4:** Recovery studies for EMT, TDF, and RPV.

Con (%)	Added amount (mg)			Amount recovered (mg)			Amount recovered (%)		
	EMT	TDF	RPV	EMT	TDF	RPV	EMT	TDF	RPV
50	10	15	1.25	9.950	15.18	1.232	99.51	101.96	98.72
75	15	22.5	1.875	14.99	22.66	1.874	99.94	100.75	99.94
100	20	30	2.5	20.20	29.53	2.526	101.03	98.44	101.04
125	25	37.5	3.125	25.14	37.64	3.062	101.27	100.38	98.00
150	30	45	3.75	30.07	44.50	3.81	100.26	98.90	101.98

**Table 5:** Precision results for EMT, TDF, and RPV.

Parameter	Sampling interval	EMT			TDF			RPV		
		Amount present (mg)	Amount present (%)	%RSD	Amount present (mg)	Amount present (%)	%RSD	Amount present (mg)	Amount present (%)	%RSD
Within-day	0 hrs	0.1987	99.35	0.75	0.2991	99.71	0.90	0.0250	98.94	0.67
	8 hrs	0.2013	100.68	0.58	0.2970	99.03	0.54	0.0247	99.06	0.51
	16 hrs	0.1996	99.84	0.37	0.2967	98.91	0.32	0.0249	99.68	0.86

Between-day	1st day	0.1997	99.85	0.61	0.2966	98.87	0.74	0.0251	100.48	0.87
	2nd day	0.1996	99.83	0.78	0.2999	99.98	0.49	0.0249	99.71	0.89
	3rd day	0.1998	99.90	0.60	0.2955	98.50	0.36	0.0250	100.03	0.57

**Table 6:** Ruggedness results for EMT, TDF, and RPV.

Parameter	EMT			TDF			RPV		
	Amount present			Amount present			Amount present		
	(gm)	(%)	%RSD	(gm)	(%)	%RSD	(gm)	(%)	%RSD
Analyst 1	0.1995	99.79	0.65	0.2985	99.50	0.95	0.0251	100.47	0.74
Analyst 2	0.2001	100.05	1.03	0.2996	99.86	1.28	0.0250	100.25	0.98

Instrument 1	0.1999	99.67	0.68	0.2981	99.38	0.79	0.0248	99.33	0.84
Instrument 2	0.2000	100.04	0.85	0.2989	99.65	0.81	0.0251	100.58	0.89

Lab1	0.2004	100.20	0.59	0.3000	100.0	0.58	0.0253	101.24	0.84
Lab 2	0.1999	99.97	0.54	0.2977	99.26	0.84	0.0248	99.38	0.83

**Table 7:** Robustness studies (by changing the wavelength).

Analyte	Wavelength (±nm)	Amount present (mg)	Amount present (%)	%RSD
EMT	239.8	0.2001	100.68	0.72
	241.8	0.2012	100.60	0.57

TDF	256.6	0.2954	98.49	0.36
	258.6	0.2963	98.78	0.56

RPV	304.6	0.0249	99.71	0.68
	306.6	0.0247	99.01	0.85

**Table 8:** Stability data of stock solutions.

DAY	EMT		TDF		RPV	
	Amount present (mg)	Amount present (%)	Amount present (mg)	Amount present (%)	Amount present (mg)	Amount present (%)
1	0.2013	100.66	0.3002	100.07	0.0255	102.11
2	0.2000	100.00	0.2971	99.06	0.0256	102.41
3	0.1992	99.64	0.2996	99.88	0.0254	101.68
4	0.1966	98.34	0.3508	101.95	0.0251	100.49
5	0.2005	100.03	0.2968	99.06	0.0244	102.41
6	0.1989	99.64	0.2992	99.88	0.0251	101.68
7	0.1966	98.34	0.3504	101.95	0.0256	100.49

**Table 9:** Assay results for commercial formulation.

Amount present (mg)	Amount present (% label claim)	Amount present (mg)	Amount present (% label claim)	Amount present (mg)	Amount present (% label claim)
EMT		TDF		RPV	
0.2004	100.20	0.2943	98.10	0.0251	100.61
0.2032	101.60	0.2940	98.01	0.0251	100.77
0.2016	100.81	0.2951	98.38	0.0250	100.32
0.1996	99.81	0.2976	99.22	0.0256	100.41
0.1989	99.46	0.2975	99.18	0.0250	100.13
0.2013	100.68	0.2968	98.94	0.0255	102.01
S.D	0.767508	S.D	0.543	S.D	0.9390
% RSD	0.764205	% RSD	0.550	% RSD	0.9293
